# Evaluating the psychometric properties of the Generic Conspiracist Beliefs Scale: a Rasch modelling approach

**DOI:** 10.3389/fpsyg.2026.1721668

**Published:** 2026-02-11

**Authors:** Andrew Denovan, Neil Dagnall, Matthew Peverell, Kenneth Graham Drinkwater, Álex Escolà-Gascón, Daniel Carter

**Affiliations:** 1 School of Psychology, Liverpool John Moores University, Liverpool, United Kingdom; 2 School of Psychology, Manchester Metropolitan University, Manchester, United Kingdom; 3 Department of Quantitative Methods and Statistics, Universidad Pontificia Comillas, Madrid, Spain

**Keywords:** conspiracy beliefs, conspiratorial ideation, Generic Conspiracist Beliefs Scale, measuring belief in conspiracy theories, multidimensional Rasch analysis, questionnaire evaluation

## Abstract

**Introduction:**

Within psychological research, the Generic Conspiracist Beliefs Scale (GCBS) has emerged as the predominant measure of conspiratorial ideation. Although studies report that the GCBS is psychometrically satisfactory (i.e., valid and reliable) and possesses a robust five-factor multidimensional latent structure, analysis has employed traditional measurement evaluation. Acknowledging this, the present paper evaluated the GCBS using Rasch analysis. Rasch analysis was necessary because it provides a more rigorous examination of measurement properties than classical test theory. This allows theorists to refine scales, develop precise measures, and advance the quality and comparability of findings across studies.

**Methods:**

The study sample comprised 2,987 UK participants (*Mage* = 32.13, *SD* = 14.18), with 1,335 males and 1,652 females. Data were collected via online survey and subjected to a series of psychometric tests to determine dimensionality and item-level performance.

**Results:**

Preliminary evaluation confirmed the presence of more than one dimension. Accordingly, the authors proceeded with multidimensional Rasch modelling, which verified that data supported the original five-factor structure. Moreover, the GCBS demonstrated satisfactory item/person fit and reliability, and items mostly displayed invariance across age (young, 18–24 years vs. older adults, 25–88 years). There were strong positive associations within GCBS dimensions; the highest was between Government Malfeasance (i.e., illegal or unethical state actions) and Control of Information (i.e., the deliberate manipulation or suppression of truth).

**Discussion:**

The presence of a multidimensional, correlated latent structure supported the notion that the GCBS assesses a range of construct breadth. Future research should evaluate GCBS item functioning across heterogeneous samples, specifically non-Western, non-English speaking, and different educational groups. Furthermore, since the Control of Information subscale demonstrated lower reliability, further refinement is essential for its effective use as a distinct index of conspiratorial ideation.

## Introduction

Though multiple self-report scales assessing conspiratorial belief exist (Drinkwater et al., 2023; Swami et al., 2017), the Generic Conspiracist Beliefs Scale (GCBS; Brotherton et al., 2013) has emerged as the principal research measure. Published citations (Goreis and Voracek, 2019) and translation of the GCBS into multiple languages (e.g., French, Lantian et al., 2016; Persian, Atari et al., 2019; Polish, Siwiak et al., 2019; Japanese, Majima and Nakamura, 2020; Spanish, Fasce et al., 2022; Serbian, Dinić et al., 2024) evidence the instrument’s predominance.

By evaluating attitudes to general statements, the GCBS appraises the thematic content of conspiracy ideation. For example, responses to ‘The government permits or perpetrates acts of terrorism on its own soil, disguising its involvement’ encapsulates features of myriad conspiracies about historical events (The Reichstag fire, 1933; Pearl Harbor, 1941; Operation Northwoods, 1962; Russian Apartment Bombings, 1999, etc.). The GCBS developed because researchers recognised the limitations of theory-based measures, which assess belief via endorsement of a specific conspiracy (e.g., London bombings, July 7, 2005; Swami et al., 2011) or subsets of theories (Drinkwater et al., 2012).

The theory-based approach derived from the presumption that belief in conspiracies is monological (Goertzel, 1994), whereby accepting one conspiracy increases the likelihood of endorsing others. While some investigators support this view (Swami et al., 2011), others argue that conspiratorial ideation is not a ‘monolithic’ mindset and that distinct domains do not necessarily align (Enders et al., 2021; Uscinski et al., 2022). Critics further argue that the monological view oversimplifies conspiratorial belief, which arises from multifarious factors.

These include inter- and intra-category differences. Inter-category variations denote differences in endorsement between discrete conspiracy theories domains of (e.g., comparing levels of belief in political vs. scientific conspiracies). Illustratively, conspiracies about ‘high-profile deaths’ (e.g., celebrity and political figures) typically receive greater support than those about ‘extraterrestrial visitation’. Conversely, intra-category variations refer to within domain differences. For example, advocacy of conspiracies about high-profile deaths fluctuates as a function of circumstances, context, and popularity (see Brotherton and French, 2014; Dagnall et al., 2015; Drinkwater et al., 2018). Noting such discrepancies, questions remain about whether specific theory responses adequately capture construct domain content and generalise to studies using different conspiracies (Hagen, 2018). Furthermore, social, political, historical, financial and temporal factors also affect conspiracy theory endorsement (Dinić et al., 2024; Leclercq et al., 2024; van Prooijen and Douglas, 2017). These factors highlight the necessity of a measurement tool that is robust across societal contexts and crisis situations.

Acknowledging this, Brotherton et al. (2013) developed the GCBS to assess belief in conspiracies via non-event-based content. To achieve this, Brotherton et al. (2013) reviewed academic and popular literature using generic descriptors (e.g., government and organizations). Then a volunteer sample, recruited via a Psychology Today blog and the Psychology of the Paranormal email list, completed the 75 items (study 1). Exploratory factor analysis (EFA) then refined the scale to 59 items organised into five factors: government malfeasance (GM) (i.e., government-related criminal conspiracies), extraterrestrial cover-up (ET) (i.e., hiding evidence of alien life), malevolent global conspiracies (MG) (i.e., secret organizations controlling world events), personal wellbeing (PW) (i.e., threats to health and freedom), and control of information (CI) (i.e., manipulation and suppression of knowledge).

Study 2 reduced the GCBS item pool to 15 items by selecting three representative statements from each of the five factors. Confirmatory factor analysis (CFA) confirmed that a five-factor correlated (vs. unidimensional) model best fitted data. The revised scale demonstrated strong reliability (internal consistency and test–retest) and criterion validity; scores correlated positively with the Belief in Conspiracy Theories Inventory (BCTI) (Swami et al., 2010), specific conspiracies (i.e., 9/11, Swami et al., 2010; and 7/7 bombings, Swami et al., 2011), and fictitious claims (i.e., the Red Bull conspiracy, Swami et al., 2011). Studies 3 and 4 extended these findings to broader populations, confirming convergent validity through associations with paranormal beliefs, delusional ideation, anomie, and lower interpersonal trust, while establishing discriminant validity via non-significant relationships with extraversion, neuroticism, sensation seeking, and emotional intelligence.

Despite data best fitting a factor correlated model, Brotherton et al. (2013) recommended using the total scale score since it captures a coherent belief system that aligns with typical conspiracy thinking. Although subsequent studies replicated the five-factor structure (Fasce et al., 2022; Siwiak et al., 2019), others reported alternative solutions. Specifically, a two-factor model comprising general and extraterrestrial conspiracist beliefs (Majima and Nakamura, 2020; Swami et al., 2017) and a three-factor model containing political, scientific, and extraterrestrial conspiracies (Atari et al., 2019). Structural discrepancies such as these reflect inherent limitations of Classical Test Theory (CTT), whereby item parameters and factor solutions lack sample invariance and are consequently sensitive to sample-specific response patterns (Embretson and Reise, 2013; Lord, 2012).

Addressing concerns about inconsistent factor replication, Drinkwater et al. (2020) re-examined the GCBS’s psychometric properties by comparing data from university students with a market research sample. Findings confirmed the five-factor model’s superior fit over one-, two-, and three-factor alternatives, with results remaining consistent across groups. Factor correlations indicated strong overarching belief in conspiracies, signifying that the GCBS effectively captures generalized conspiracist thinking. Additionally, the scale demonstrated high internal reliability; convergent validity was also established via significant correlations with proneness to reality-testing deficits and subjective-intuitive thinking (Drinkwater et al., 2020).

Overall, research findings indicate that the GCBS is a reliable measure. However, although possessing a robust higher-order factor structure, the composition and stability of GCBS subscales appear sensitive to contextual and sample-specific variation (Atari et al., 2019; Majima and Nakamura, 2020; Swami et al., 2017). Consistent with this pattern, the weak fit of a unidimensional model suggests that although GCBS factors are strongly interrelated, endorsement levels vary across subscales. For example, in the Brotherton et al. (2013) second study, GM shared greater variance with MG (66%), PW (75%), and CI (55%) than ET (31%), highlighting that not all conspiracy beliefs receive equal support. This inconsistency poses challenges since researchers often use the GCBS as a global measure (e.g., Cosgrove and Murphy, 2023).

In this context, collapsing multifaceted beliefs into a single global score obscures meaningful variability across content domains (Douglas and Sutton, 2023). Moreover, summation potentially oversimplifies the construct and may produce inconsistent or misleading findings. Specifically, correlates such as political orientation or trust may relate differently to distinct conspiracy domains. Global scores cannot capture these nuances. Despite these concerns, the GCBS remains highly influential as evidenced by the fact that researchers have recently developed a shortened, unidimensional version of the instrument for use in extended self-report batteries (see GCB-5: Dagnall et al., 2023; Kay and Slovic, 2023); this draws on the highest loading item from each of the five dimensions. To provide even greater expediency in research settings where survey length is highly constrained, researchers have developed a single-item measure for capturing generalized conspiracist belief (Lantian et al., 2016).

To address these structural discrepancies and extend the psychometric evaluation of the GCBS, the present study employed Rasch analysis (RA). Whereas confirmatory factor analysis (CFA) is well established for testing latent structure within a given sample, RA offers a complementary approach grounded in the principles of fundamental measurement. Unlike CFA, which evaluates how well a model fits observed data, RA assesses the extent to which data conform to a prescriptive measurement model, an approach that some theorists contend affords greater rigor and interpretability (Bond and Fox, 2013; Tennant and Conaghan, 2007).

This distinction is evident in how each approach handles data levels. CFA typically relies on Classical Test Theory (CTT) assumptions about the interval-level properties of observed data, whereas RA explicitly tests whether ordinal Likert-scale responses transform into a linear, interval-level metric (logits). Unlike the correlational emphasis of CFA, RA assesses the extent to which data conform to a prescriptive mathematical model grounded in fundamental measurement principles, operating at the item level to identify responses that deviate from expected patterns or difficulty hierarchies. This approach allows researchers to evaluate whether individual items contribute meaningfully to the measurement of the latent trait. This is important as items that respondents endorse too frequently or rarely can undermine the scale’s discriminatory power.

A key strength of RA is the ability to establish local item independence by identifying dependencies through misfit. Additionally, RA provides infit and outfit indices to detect excessive response randomness or predictability. Furthermore, RA allows for the detection of Differential Item Functioning (DIF), which occurs when item difficulty varies as a function of group characteristics (e.g., age, gender) rather than the underlying latent trait. Detecting such systematic error prevents scale bias and ensures measurement invariance. In the present study, because research indicates that conspiratorial endorsement may vary by age, RA was used to assess potential DIF between younger and older adults (Byrne et al., 2024; Jolley et al., 2021).

Another positive feature of RA is the ability to place person measures and item measures on a unified interval scale. This enables comparison between specific item difficulty (i.e., likelihood of endorsement) and respondent trait levels (i.e., facilitates the assessment of scale targeting). Scale targeting refers to the extent to which the range of item difficulty in a scale matches the range of the latent trait (e.g., conspiracy belief) within the study population; a well-targeted scale ensures that items are neither too easy nor too difficult for the participants, thereby maximizing measurement precision. While CFA provides important item-level information via factor loadings and thresholds, it does not provide this shared person and item parameter metric. Furthermore, RA facilitates the identification of individuals whose response patterns deviate from model expectations (e.g., through infit and outfit statistics), offering a standardized approach to person-misfit detection. In addition to these gains, the Rasch model is a probabilistic framework designed for ordinal data, such as Likert scales, and does not require the strict assumption of multivariate normality associated with maximum likelihood estimation in CFA.

As a consequence of these features, Rasch models ensure measurement properties remain stable across samples. This enhances generalizability compared to CFA, which produces sample-dependent estimates. RA also tests for unidimensionality using item fit statistics, whereas CFA relies on factor loadings and goodness-of-fit indices that may fail to detect multidimensionality.

A further advantage of RA is relative sample size efficiency compared to CFA. While CFA typically requires large samples to ensure stable parameter estimates and the reliability of fit indices (e.g., N > 500), RA provides stable item calibrations with smaller cohorts. Linacre (1994) demonstrated that a sample of 243 participants was sufficient to provide 99% confidence that no item calibration deviated by more than 0.5 logits from its stable value. Furthermore, even smaller samples ranging from 100 to 150 can yield preliminary calibrations for instrument development (Wright and Tennant, 1996). This makes RA a robust alternative for psychometric evaluation when large-scale data collection is constrained.

Concomitantly, because RA transforms raw ordinal scores into interval-level measures and provides item parameters (i.e., difficulty and discriminatory power), the procedure makes more meaningful comparisons than the factor loadings used in CFA. Based on psychometric GCBS literature (Brotherton et al., 2013; Drinkwater et al., 2020) and the capabilities of Rasch analysis, the authors hypothesized that, consistent with prior CFA-based research, Rasch analysis would support the notion that the GCBS is multidimensional rather than unidimensional (H1). Moreover, GCBS items should demonstrate acceptable item fit, confirming the psychometric quality of the existing scale items (H2). Additionally, although younger adults should demonstrate greater propensity to endorse conspiracy theories, the scale will remain robust, with DIF found for no more than a negligible number of items, suggesting measurement equivalence across age groups (H3).

## Methods

### Participants

To produce a large, heterogeneous sample the researchers merged three individual data sets from ongoing projects and doctoral work. Within these scholarly activities, respondents completed the GCBS alongside other psychological measures. All participants resided in the UK and were at least 18 years old. The combined sample comprised 2,987 participants (*Mage* = 32.13, *SD* = 14.18, range = 18 to 88); 1,335 males (*Mage* = 36.21, *SD* = 14.51, range = 18 to 75), and 1,652 females (*Mage* = 28.82, *SD* = 13.01, range = 18 to 88). The researchers produced this data set by merging independent datasets collected between 2021 and 2023. While the original studies investigated psychological correlates of conspiratorial ideation, this paper focused on the internal measurement properties of the GCBS. To prevent multiple responses, instructions asked respondents to specify whether they have participated within similar studies. Respondent recruitment occurred through university networks and advertisements on social (e.g., Facebook) and professional networking platforms (e.g., LinkedIn). Participation was voluntary, and the researchers provided no financial compensation nor academic credit for survey completion.

### Measures

#### Generic Conspiracist Beliefs Scale

The Generic Conspiracist Beliefs Scale (Brotherton et al., 2013) consists of 15 statements (e.g., ‘A small, secret group of people is responsible for making all major world decisions, such as going to war”) that evaluates general conspiratorial ideation across five distinct conceptual domains: Government Malfeasance (i.e., illegal or unethical state actions); Malevolent Global Conspiracies (i.e., control of world events by secret groups); Extraterrestrial Cover-Up (i.e., suppression of evidence regarding alien contact); Personal Wellbeing (i.e., secret concerns regarding public experimentation or technology); and Control of Information (i.e., the deliberate manipulation or suppression of truth). Participants rate their agreement to items using a 5-point Likert scale (1 = definitely not true to 5 = definitely true). The scale encompasses five subscales (see Introduction) and total scores reflect overall levels of conspiratorial ideation; higher scores indicate stronger conspiratorial beliefs.

#### Procedure

Individuals who responded to the call for participants accessed the Participant Information Sheet through a web link. This document detailed the study’s intention, objectives, and ethical considerations. Those who agreed to participate provided consent before proceeding to the survey, which included demographic questions (such as gender and age) and measurement instruments. To minimize potential order effects, the researchers used the Qualtrics randomizer to vary scale presentation order. Although participants completed an array of measures as part of the original data collection phases (e.g., assessments of paranormal belief and cognitive style), for the purpose of the present psychometric evaluation the researchers only extracted GCBS responses. After completing the survey, participants received a debrief. The debriefing reiterated the study aims, reaffirmed that items were generic examples used for psychological assessment, and restated the contact information for the lead researcher and institutional ethics committee.

Since the study employed a cross-sectional design, the researchers used remedies to reduce common method variance, evaluation apprehension, and social desirability bias (see Dagnall et al., 2022a, 2022b). Specifically, both general and scale-specific instructions highlighted the distinctiveness of subsections, creating psychological separation between scales and encouraging thoughtful responses (Krishnaveni and Deepa, 2013). The directions asked participants to read each statement carefully, work at their own pace, respond to every item, and keep in mind that no answers counted as right or wrong.

### Analytical plan

Following data screening, the researchers performed Rasch analysis (RA) on GCBS data. Focusing on a Principal Components Analysis (PCA) of residuals, the investigators assessed unidimensionality using Winsteps 3.81 (Linacre, 2014). Eigenvalues >2.0 indicate that an extracted dimension is meaningful. Such values are inconsistent with the unidimensionality assumption of the Rasch model (Linacre, 2012).

The researchers tested the assumption of local independence using Yen’s Q3 statistic, which identifies instances where item responses correlate beyond what the underlying latent trait would predict. Multidimensional RA of the GCBS used ConQuest version 5.34 (Adams et al., 2023). Analysis used the Monte Carlo method to simulate data for evaluating model performance, in conjunction with marginal maximum likelihood estimation to estimate item and person parameters.

Since GCBS items use a polytomous format with more than two response categories, analyses employed the Partial Credit Model (PCM). This model allows each item to have its own scoring structure, making it well suited to varying levels of endorsement across response options. Following inspection of response scale functioning, item fit was evaluated using Infit and Outfit Mean Square (MNSQ) statistics, which quantify the degree of misfit between the observed data and model expectations. Values between 0.5 and 1.5 are ideal (Linacre, 2025), and values between 1.5 and 2.0 only impede measurement quality if multiple occurrences exist (Wright and Linacre, 1994).

An item-person map assessed how well the difficulty of each item matched the levels of the trait (e.g., belief in conspiracy theories) shown by the respondents. This visual tool located items and individuals on the same scale, allowing researchers to determine whether items are appropriately targeted (i.e., effectively measure the full range of beliefs present in the sample). Further analysis assessed person reliability indices, differential item functioning (DIF) (i.e., comparison between younger and older adult responses), and explored construct structure by examining correlations among latent dimensions. Existing research indicating that younger (vs. older) participants are more prone to endorsing conspiracy theories informed the decision to assess age (Byrne et al., 2024; Jolley et al., 2021).

Consistent with the theoretical distinction between emerging and established adulthood (Arnett, 2000, 2007), the researchers categorized participants into two groups: young adults (18–24 years) and older adults (25–88 years). This dichotomy was psychometrically appropriate because RA remains robust to within-group heterogeneity when group definitions are theoretically grounded and sample sizes sufficient (Bond and Fox, 2013; Linacre, 2012). Using these broad groupings maximized statistical power and ensured stable parameter estimation, both critical for reliable DIF analysis (Linacre, 2012; Scott et al., 2009). Although the researchers considers alternative strategies, such as quartile splits, these would have reduced power and interpretability without affecting the substantive pattern of results. Moreover, distinguishing emerging from established adulthood aligned with prior age invariance research (e.g., Schwartz et al., 2005; Tucker-Drob, 2009).

## Results

Data screening found acceptable univariate normality (i.e., skewness and kurtosis values between −3.0 and +3.0). Initial assessment of GCBS unidimensionality, using PCA residuals, produced a contrast eigenvalue of 3.3, indicating the presence of multiple dimensions. Furthermore, Yen’s Q3 revealed 10 correlations exceeded 0.20 (the absolute value representing local dependence) (Yen, 1984). This further indicated multidimensionality, suggesting that more than one latent trait influenced item responses. Accordingly, calibration of GCBS dimensions occurred, this focused on the established five-factor model by Brotherton and French (2014) (i.e., comprising Government Malfeasance, Malevolent Global Conspiracies, Extraterrestrial Cover-up, Personal Wellbeing, and Control of Information). Response scale scrutiny revealed that step calibrations monotonically increased (albeit below five logits) across all items (Hawkins et al., 2023), indicating that the 5-point rating scale was appropriate. Outfit and Infit MNSQ for all items were within Linacre’s (2025) optimum range of 0.5 and 1.5 (see Table 1), which indicates appropriate data-model fit for the five-dimensional model.

**Table 1:** Psychometric properties of the Generic Conspiracist Beliefs Scale at the item level.

Item	Factor	Infit MNSQ	Outfit MNSQ	Difficulty
1. The government is involved in the murder of innocent citizens and/or well-known public figures, and keeps this a secret.	Government Malfeasance	0.98	1.0	−0.04
6. The government permits or perpetrates acts of terrorism on its own soil, disguising its involvement.	Government Malfeasance	0.99	1.0	0.26
11. The government uses people as patsies to hide its involvement in criminal activity.	Government Malfeasance	0.96	0.97	−0.22
2. The power held by heads of state is second to that of small unknown groups who really control world politics.	Malevolent Global Conspiracies	1.03	1.04	−0.15
7. A small, secret group of people is responsible for making all major decisions, such as going to war.	Malevolent Global Conspiracies	1.03	1.04	0.15
12. Certain significant events have been the result of the activity of a small group who secretly manipulate world events.	Malevolent Global Conspiracies	0.86	0.89	0.01
3. Secret organizations communicate with extraterrestrials, but keep this fact from the public.	Extraterrestrial Cover-up	0.97	1.01	0.26
8. Evidence of alien contact is being concealed from the public.	Extraterrestrial Cover-up	1.0	1.02	−0.36
13. Some UFO sightings and rumours are planned or staged in order to distract the public from real alien contact.	Extraterrestrial Cover-up	0.92	0.95	0.10
4. The spread of certain viruses and/or diseases is the result of the deliberate, concealed efforts of some organization.	Personal Wellbeing	0.92	0.92	−0.03
9. Technology with mind-control capacities is used on people without their knowledge.	Personal Wellbeing	1.08	1.08	0.21
14. Experiments involving new drugs or technologies are routinely carried out on the public without their knowledge or consent.	Personal Wellbeing	1.0	1.0	−0.18
5. Groups of scientists manipulate, fabricate, or suppress evidence in order to deceive the public.	Control of Information	0.87	0.88	0.36
10. New and advanced technology which would harm current industry is being suppressed.	Control of Information	0.94	0.94	0.22
15. A lot of important information is deliberately concealed from the public out of self-interest.	Control of Information	1.05	1.06	−0.57

**Figure 1:**
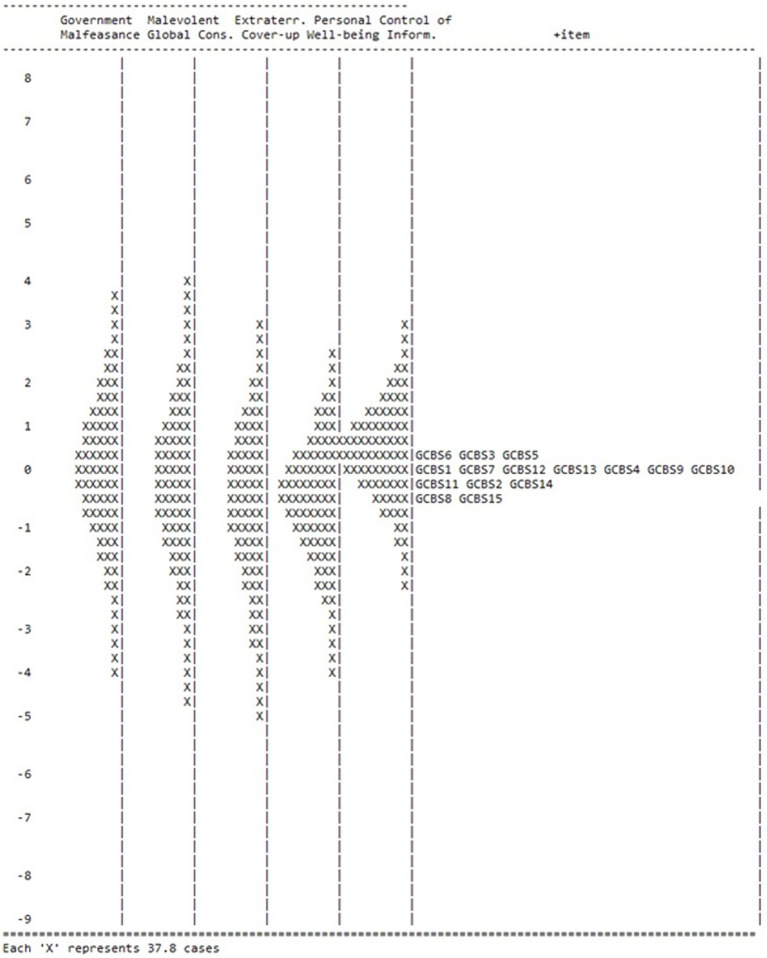
Item-person map for the GCBS. The participants are on the left and more able participants are located at the top of the map. Items are located on the right (under ‘+item’) and more difficult items are located at the top of the map. Each ‘X’ represents 37.8 cases. Interpretation includes comparing the distribution of person abilities (left side, low to high) against item difficulties (right side, easy to hard) on the same vertical logit scale; alignment indicating good targeting.

The Rasch model identifies item difficulty in relation to a sampled population. Explicitly, an item’s difficulty (or ease) represents a specific point on a continuum that, when compared to a person’s ability, determines the likelihood of a correct response. Easier items have a higher probability of being answered correctly by most people, whereas harder items require greater ability. Difficulty and ability are measured in the same units (logits) on a unified scale. This model transforms raw scores into meaningful measures, placing people and items on the same scale. This process reveals gaps or clusters in measurement and enables precise evaluation of test quality (Kean et al., 2018). The item-person map (Figure 1) presented this difficulty continuum, and specified that participants found item 8 (Extraterrestrial Cover-up; ‘Evidence of alien contact is being concealed from the public’) and 15 (Control of Information; ‘A lot of important information is deliberately concealed from the public out of self-interest’) the easiest, and items 6 (Government Malfeasance; ‘The government permits or perpetrates acts of terrorism on its own soil, disguising its involvement’), 3 (Extraterrestrial Cover-up; ‘Secret organizations communicate with extra-terrestrials but keep this fact from the public’), and 5 (Control of Information; ‘Groups of scientists manipulate, fabricate, or suppress evidence in order to deceive the public’) the most challenging to endorse.

Item difficulty verified this (see Table 1); scores ranged from −0.57 (item 15), indicating it was easy to endorse, to 0.36 (item 5) logits, suggesting it was difficult to approve. Although items clustered closely, the Rasch person measure means differed in each GCBS dimension, additionally supporting multidimensionality (Table 2). The lowest mean existed for Extraterrestrial Cover-up, indicating that respondents did not strongly endorse the subscale.

**Table 2:** Mean of person measure and associations between GCBS dimensions.

GCBS dimension	Person mean	Government malfeasance	Malevolent global conspiracies	Extraterrestrial cover-up	Personal wellbeing	Control of information
Government malfeasance	0.09		3.16	2.66	2.20	1.70
Malevolent global conspiracies	0.25	0.83		3.12	2.64	1.76
Extraterrestrial cover-up	−0.77	0.73	0.74		2.40	1.49
Personal wellbeing	−0.48	0.86	0.89	0.85		1.25
Control of information	0.44	0.90	0.80	0.71	0.84	

The top diagonal includes covariances; the bottom diagonal includes correlations.

In terms of subscale efficiency, Person Separation Reliability was 0.78 for Government Malfeasance, 0.80 for Malevolent Global Conspiracies, 0.76 for Extraterrestrial Cover-up, 0.71 for Personal Wellbeing, and 0.66 for Control of Information. Control of Information indicated lower measurement precision due to falling below the recommended 0.7 cutoff. Item Separation Reliability scores (i.e., how effectively the sample could differentiate items within the GCBS) were 0.91 for Government Malfeasance, 0.92 for Malevolent Global Conspiracies, 0.90 for Extraterrestrial Cover-up, 0.71 for Personal Wellbeing, and 0.86 for Control of Information, reflecting good reliability.

Differential item functioning (DIF) analyses revealed that the older age group scored lower than the younger age group. Moreover, item 1 (Government Malfeasance; ‘The government is involved in the murder of innocent citizens and/or well-known public figures and keeps this a secret’) showed evidence of DIF (greater than 0.5 logits). The remaining items showed no evidence of DIF (i.e., all below 0.5 logits). Though scale modification was not the focus, the authors hope this information will prove useful for future researchers. Associations between GCBS dimensions ranged from 0.71 to 0.90 (Table 2). Corrected for error, correlations were free from measurement ‘noise’ (Briggs and Wilson, 2003). All dimensions correlated highly.

## Discussion

The outcomes of Rasch analysis (RA) supported hypotheses regarding the measurement properties of the Generic Conspiracist Belief Scale (GCBS). Specifically, they confirmed H1 (Multidimensionality) by verifying that the instrument assesses highly correlated yet conceptually distinct dimensions of conspiratorial beliefs. Psychometric robustness extended to individual item performance (H2), with all 15 items demonstrating optimal fit statistics. Finally, as predicted by H3, the GCBS remained largely invariant across age groups. Although younger participants endorsed items more readily, one item exhibited differential item functioning (DIF). A detailed evaluation of these findings follows.

By stringently testing unidimensionality and item-level performance, the present paper extended psychometric evaluation of the Generic Conspiracist Belief Scale (GCBS). Regarding latent structure, analyses confirmed GCBS multidimensionality. This concurred with preceding work using classic CFA testing, which reported that GCBS subscales assess highly related but discrete facets of conspiracy ideation (Brotherton et al., 2013; Drinkwater et al., 2020). In terms of dimensions, this outcome aligned with Drinkwater et al. (2020) who reported that the correlated five-factor solution (vs. one, two, and three-factor models) provided best data fit.

Structural inconsistencies noted in prior studies (i.e., two-factor, Majima and Nakamura, 2020; Swami et al., 2017; and three-factor, Atari et al., 2019 models) likely stem from the use of exploratory or confirmatory factor analysis within heterogeneous samples. Unlike these CTT-based approaches, which are often sample-dependent, the Rasch model evaluates the invariant measurement properties of the items. By transforming ordinal responses into interval-level logits, RA demonstrates that the five factors represent distinct difficulty hierarchies that remain stable regardless of the sample’s average level of belief. Consequently, by showing that the five-factor multidimensional model is the most psychometrically robust representation of the construct, the present findings provide a more definitive answer to structural debates.

While Rasch analysis confirmed that these five subscales provide a psychometrically robust framework, it is important to consider whether these domains accurately capture the contemporary conspiracy landscape. The GCBS was purposefully designed to avoid the temporal and geographic limitations of event-based measures. However, modern conspiratorial ideation reflects increased medical, scientific, and technological scepticism that the GCBS Personal Wellbeing and Control of Information dimensions only partially or indirectly addresses. For instance, recent global discourse has highlighted themes such as biosecurity and digital surveillance. While the GCBS items regarding ‘secret experiments’ and ‘suppressed technology’ touch upon these areas, future iterations of the scale might benefit from items that directly reflect these emerging concerns. Nonetheless, the present five domains remain relevant as they map onto the psychological motives of institutional distrust and detection of hidden agency.

Accordingly, consistent with typical scale use, high factor correlations suggest that the GCBS can provide a valid global index of conspiratorial belief. However, researchers should interpret outcomes with caution. Though dimensions are strongly related, they represent distinct conceptual domains that may relate differently to external criteria. The presence of this multidimensional correlated latent structure indicates that the GCBS assesses a significant breadth of construct content. This is important since conspiratorial ideation encompasses a wide range of ideas, cognitions, and perceptions (e.g., existence of malevolent groups, distrust of official explanations, detection of hidden patterns, and resistance to contrary evidence). Therefore, rather than assuming a total score is always the most informative metric, investigators should consider whether subscale-specific analysis more appropriately captures the nuanced psychological or political drivers of different conspiracy types. From a practical perspective, these findings support the use of the GCBS as a psychometrically robust and expedient measure (Brotherton et al., 2013; Dinić et al., 2024; Drinkwater et al., 2020). This approach is pertinent given criticisms of monolithic conceptualizations and increased emphasis on content-specificity within the conspiracy literature.

Application of RA provided a nuanced evaluation of GCBS item functioning by estimating item difficulty relative to respondent endorsement patterns. This revealed a hierarchy of belief that aligned with broader conspiracy prevalence literature (e.g., Oliver and Wood, 2014), where more generalized suspicions are more readily ratified than theories involving extreme government violence. For instance, item 15 (‘A lot of important information is deliberately concealed from the public’) was among the easiest to endorse. However, from a measurement perspective, the ease of endorsement for item 15 may stem from its linguistic vagueness. As noted by critics (e.g., Sutton and Douglas, 2020), participants endorse items for a wide variety of reasons. These range from legitimate concerns about government transparency to deep rooted distrust. This breadth potentially reduces item discriminative power.

In contrast, participants found items 6 (‘The government permits or perpetrates acts of terrorism on its own soil’) and 3 (‘Secret organizations communicate with extra-terrestrials’) significantly more difficult to ratify. These items represent reflect conspiratorial thinking that deviates from social norms and perceived plausibility. The fact that endorsement of these items requires a higher level of latent conspiratorial ideation confirms that the GCBS captures a spectrum of beliefs ranging from mundane scepticism to extreme ideation. This difficult hierarchy demonstrates that the scale assesses a graduated continuum of ideological commitment.

Thematically, these items assess institutional deception and hidden agendas by powerful entities (i.e., high-stakes conspiracy thinking; conviction that large, powerful entities engage in covert, deceptive actions that shape society). Despite these differences, response patterns failed to reveal poor item functioning. Rather, the observed variation reflected differing levels of belief intensity across conspiracy themes, thereby supporting the scale’s capacity to distinguish respondents based on the extremity of their conspiracy beliefs.

An important advantage of RA is the procedure’s ability, by placing item difficulty and person ability on a shared logit scale, to provide item-level precision. This is important since it affords interpretable estimates of construct alignment. The distribution of person measures across GCBS subscales also supported previous findings of multidimensionality. A feature of which was low observed endorsement of the Extraterrestrial Cover-up dimension. Reliability indices for person and item separation exceeded commonly accepted thresholds, indicating strong internal structure and high measurement precision across subscales.

However, the Control of Information subscale demonstrated lower reliability (item separation and person reliability) compared to the other dimensions. This weaker performance may arise from the conceptual breadth of the subscale, which ranges from specific (i.e., scientific fraud, item 5) to generalized claims (i.e., information suppression, item 15). The indistinctness of the latter allows for differing interpretations, which can introduce measurement error and reduce internal consistency. To refine this subscale, future research should replace broader, more equivocal items with statements that target particularly forms of information control. Such refinements would decrease response variance and improve the ability of the subscale to precisely locate individuals on the latent trait of conspiratorial ideation.

The younger age group endorsed GCBS items more readily, but only one item showed substantial differential item functioning (DIF) (item 1: ‘The government is involved in the murder of innocent citizens and/or well-known public figures and keeps this a secret’). However, given this was a single item, the difference was not likely to unduly influence measurement. Nonetheless, feature refinement of item 1 is advisable to enhance psychometric performance. This instance highlights that a strength of RA is the ability to detect response biases overlooked by traditional psychometric approaches. Collectively, findings highlighted the GCBS’s overall psychometric robustness and the value of RA in confirming the appropriateness of latent response structures and guiding future scale development.

### Limitations

While RA indicated GCBS multidimensional, it is important to acknowledge that strong positive correlations between subscales suggest an underlying general factor, which is indicative of, but does not meet the criteria for unidimensionality. The potential presence of a dominant dimension further supports the conclusion that overall scores provide a psychometrically valid and conceptually meaningful. Noting this, to identify potential superordinate factors or sub-dimensions that underpin the observed multidimensionality, subsequent investigations should explore the hierarchical structure of conspiratorial beliefs.

Within the present paper, the authors restricted differential item functioning (DIF) analysis to age. This was because the researchers did not collect data concerning other key demographic variables (e.g., education level and socioeconomic status) during the recruitment phase. While gender was recorded, the primary focus was on age differences in conspiratorial ideation, hence the prioritization of age-based invariance. Although this was an important contribution to the psychometric validation of the GCBS, the omission of broader DIF analysis remains a limitation. Future studies should consider DIF as a function of other demographic variables (i.e., gender, education level, and socioeconomic status). These analyses will further understanding by establishing whether GCBS item endorsement varies as a function of subgroup membership. Using this broad set of demographic variables will ensure measurement validity and fairness when the GCBS is employed across diverse populations. Identifying differences will inform the development of equitable and accurate measures of conspiratorial beliefs.

In this article, despite the GCB-5 comprising a subset of GCBS items, the researchers constrained RA to the full 15-item form. This is a limitation, because the study did not verify whether the abridged GCB-5 retains the psychometric properties (e.g., unidimensionality and fit) of the parent measure. While test users often assume that scale short forms inherit the properties of long versions, performance can vary due to context effects. Consequently, though the current findings validate the 15-item structure, the GCB-5 requires independent validation to confirm its performance as a separate measure. Specifically, from a Rasch perspective, it is necessary to establish that the reduced item set maintains a stable item difficulty hierarchy and sufficient person separation without the contextual support of the full item bank. This is necessary because use of GCB-5 is likely to increase as awareness of the instrument increases, especially when investigators are assessing conspiratorial belief within lengthy multiple construct test batteries.

Beyond study-specific limitations, it is important to acknowledge general procedural issues that potentially restrict the usefulness of outcomes. A common concern with self-report measures such as the GCBS is vulnerability to potential response biases (i.e., social desirability, evaluation apprehension, and demand characteristics). Specifically, participants might consciously or unconsciously alter their responses to align with perceived social norms or expectations. This is particularly pertinent to the study of conspiratorial ideation, which involves non-normative or controversial beliefs that participants may be reluctant to endorse for fear of being perceived as irrational. In a Rasch framework, such biases are problematic as they can manifest as misfitting person patterns, where respondents provide idiosyncratic strings that distort item difficulty estimates. For example, a respondent seeking to appear socially conforming might selectively suppress their endorsement of ‘common’ suspicions (low-difficulty items), while paradoxically affirming more extreme, high-stakes theories that align with their worldview. Such non-Guttman response patterns generate high outfit statistics, whereby a respondent’s profile fails to align with the model’s predicted hierarchy of belief. Though the researchers used procedural remedies (e.g., anonymity, instructions for honest responding) and data screening to moderate possible effects, complete elimination in self-report is difficult. Consequently, the potential for subtle, undetected biases remains and can compromise the accuracy and generalizability of findings.

Future research could address this limitation by incorporating explicit bias-detection scales within the questionnaire. This technique reflects the approach used by the Marlowe-Crowne Social Desirability Scale, which comprises items such as ‘I always try to practice what I preach’. Identifying a participant’s tendency to respond in a socially desirable manner, would enable researchers to identify and statistical control biased responses. This would allow for the empirical identification of whether “misfitting” person statistics in the Rasch model are driven by such biases. Additionally, using multiple time points to assess response consistency would reduce potential self-report bias.

Additionally, the use of multiple time points ensures response reliability and enables the observation of changes in conspiratorial beliefs within individuals over extended periods. This is necessary advance since the current study used a cross-sectional design, where the researchers assessed conspiratorial beliefs at a single point in time. The issue with a cross-sectional design is that collected data provides only an indicator of belief and investigators cannot use outcomes to infer causal relationships. Longitudinal data would further allow for the assessment of temporal invariance, confirming that the GCBS item hierarchy remains stable across multiple assessment waves. Establishing such stability is a prerequisite for measuring true change, as it ensures that fluctuations in item difficulty or shifts in construct meaning over time do not confound observed shifts in person ability (i.e., latent conspiratorial ideation).

Though, to ensure the generalisability of findings, this study assessed a large, heterogeneous sample, it is advisable to replicate the study with independent samples. This is important for confirming the stability of the item difficulty hierarchy across different cultural and language contexts. Thus, future research should include diverse and traditionally hard-to-reach populations via community-based sampling and international collaborations. Such efforts will enhance the external validity of findings and help to establish measurement invariance across diverse person-ability groups. Within a Rasch framework, this is a prerequisite for performing meaningful cross-national comparisons, as it ensures that the relative difficulty of endorsing specific conspiratorial themes (e.g., government malfeasance vs. extraterrestrial cover-ups) remains constant across different global populations. Establishing this parameter invariance would confirm the GCBS as a universal instrument for measuring conspiratorial ideation.

## Data Availability

The raw data supporting the conclusions of this article will be made available by the authors, without undue reservation.
